# Laparoscopic Roux-en-Y Gastric Bypass with the Maestro™ System: a Novel Collaborative Robotic Platform

**DOI:** 10.1007/s11695-026-08681-7

**Published:** 2026-05-14

**Authors:** Dan Azagury, Fabrice Leclerc, Elisa Reitano, Marius Nedelcu, Henry Mercoli

**Affiliations:** 1 Stanford University School of Medicine, Stanford University, Stanford, USA; 2 Digestive Surgery, Polyclinique de Franche Comté, Besançon, France; 3 IHU mix Surg, Hôpitaux Universitaires de Strasbourg, Strasbourg, France; 4 ELSAN, Hopital privé Bouchard, Marseille, France

**Keywords:** Gastric bypass, Robotic surgery, Maestro assistance, Artificial intelligence

## Abstract

**Background:**

Laparoscopic Roux-en-Y gastric bypass (LRYGB) is a cornerstone of bariatric surgery, yet its complexity poses challenges in visualization and retraction, often compounded by variability in surgical assistance. The Maestro™ is an Artificial Intelligence (AI)-powered surgical platform designed to elevate minimally invasive surgical performance and technique while keeping the surgeon at the patient’s bedside during the procedure.

**Objective:**

This study aims to assess the effects of this innovative platform by comparing it with traditional L-RYGB.

**Methods:**

This retrospective, matched cohort study included patients who underwent Maestro™ (MA-RYGB) and traditional laparoscopic Roux-en-Y gastric bypass (L-RYGB) at a single institution. The 54 Maestro™-assisted RYGB procedures were included prospectively. Each case was retrospectively matched in a 1:1 ratio with a baseline laparoscopic RYGB performed by the same surgical team. Operative time, in-room time, and related variables were recorded for both groups. Statistical Process Control (SPC) charts were generated to evaluate the process stability and variability for operative times.

**Results:**

The Maestro™ demonstrated a 19.3% reduction in operative time: MA-RYGB 62.9 min vs.75.1 min (*p* < 0.001). Operative times appeared more stable across consecutive cases in the Maestro cohort compared with conventional laparoscopy. Postoperative outcomes were comparable. No device-related complications or conversions occurred.

**Conclusion:**

Maestro™ reduces procedure duration and was associated with a more stable operative course, without compromising safety. The use of the Maestro™ and may help standardize outcomes and operating room performance. This initial work suggests important implications for operative quality and efficiency with this promising new technology.

## Key Points

Maestro™-assisted RYGB was associated with shorter operative time.

The use of the Maestro™ suggested improved operative efficiency and consistency compared to L-RYGB.

Maestro™ reduces procedure duration, and may help standardize outcomes and operating room performance.

## Introduction / Purpose

Bariatric surgery is widely recognized as the most effective intervention for sustained weight loss and metabolic improvement in individuals with severe obesity. Laparoscopic Roux-en-Y gastric bypass [1, 2] is one of the most commonly performed bariatric procedures worldwide. L-RYGB remains one of the most technically demanding interventions due to both its procedural complexity and its reliance on optimal visualization and exposure throughout the operation. The quality of endoscopic control, tissue retraction, and overall workflow efficiency are critical factors influencing outcomes, operative time, and surgical ergonomics. A persistent challenge in laparoscopic bariatric procedures is the inherent variability introduced by human assistants. Inconsistent laparoscope handling, unanticipated changes in the field of view, hand tremor, fatigue, and suboptimal communication between the surgeon and assistant can negatively affect performance and extend operative times. The rate of complications following L-RYGB is closely related to the surgeon’s experience and institutional learning curve. Structured training, adherence to standardized techniques, and high-volume centers are critical factors in minimizing risks and optimizing patient outcomes [3, 4].

**Fig. 1:**
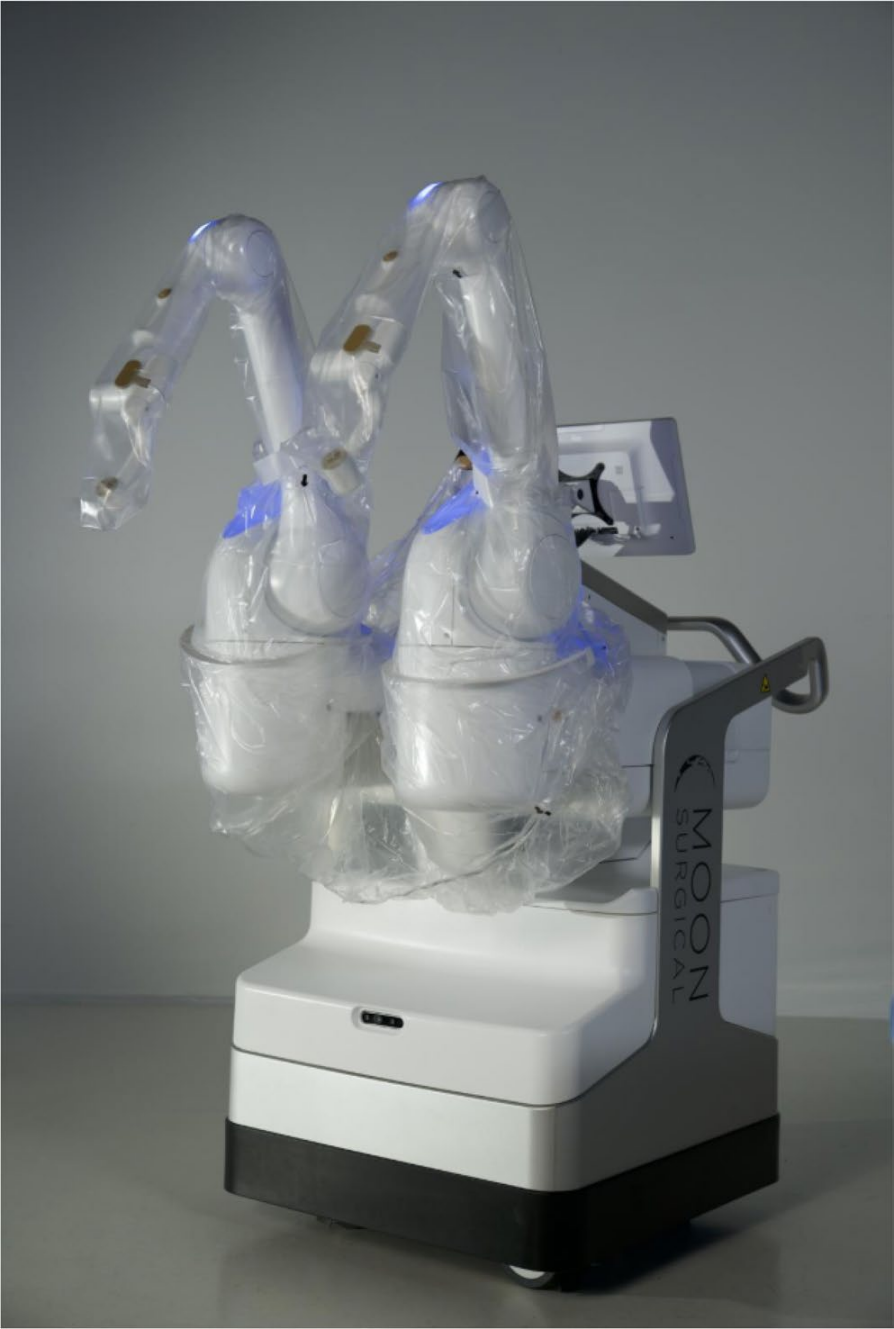
Maestro Device

Collaborative platforms represent a promising evolution of minimally invasive techniques with the potential to standardize key aspects of the operative environment and enhance overall surgical performance [5, 6]. The Maestro™ (Moon Surgical, Paris, France) is a new generation of intelligent, compact bedside platforms (Fig. 1). The system consists of a compact, intelligent cart equipped with a GPU enabling AI-driven computer vision analysis, and two responsive arms that glide smoothly and stay exactly where you leave them. These arms function as an additional pair of hands for the surgeon: one provides precise laparoscope control and stability, while the second delivers active dynamic assistance by retracting tissues and maintaining optimal exposure of the operative field. Unlike large form factor robotic systems that separate the surgeon from the patient and require bedside staff for support, Maestro preserves a bedside workflow: the surgeon remains scrubbed-in and physically present at the patient’s side, maintaining tactile feedback and empowered autonomy over the procedure allowing the surgeon to become their own assistant [7, 8]. These systems have shown promising results in general laparoscopic surgery, with shorter learning curves and better operative metrics when compared with larger, more expensive platforms [9, 10]. Despite the growing interest in this new AI-enabled collaborative platform, there is limited comparative evidence assessing its impact on performance in complex procedures like L-RYGB. This study aims to address that gap by analyzing the operative performance and intraoperative variability of Maestro-assisted RYGB (MA-RYGB) versus a matched conventional L- RYGB cohort. Outcomes measured include operative and in-room times, device-related and overall complications rates, and operative time variability.

## Materials and Methods

This was a single-center, comparative study conducted between January 2023 and January 2025. The study was conducted in a bariatric unit with two senior surgeons experienced in advanced laparoscopic bariatric surgery, each having performed several thousand laparoscopic procedures before the study period. All procedures were performed by the same surgeons using standardized operative techniques.

**Fig. 2:**
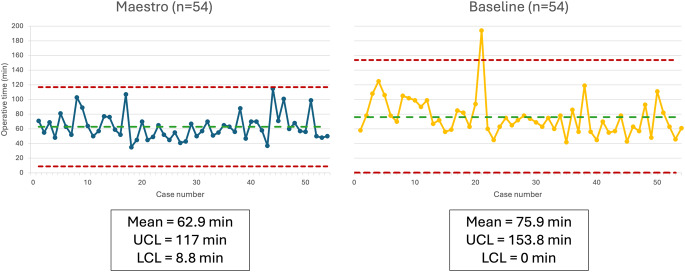
SPC Charts for operative time – Maestro vs Baseline (54 Matched cases)

The 54 Maestro™-assisted RYGB procedures were included prospectively between November 2023 and January 2024. Each case was retrospectively matched in a 1:1 ratio with a baseline laparoscopic RYGB performed in 2023 by the same surgical team. Each MA-RYGB case was matched to a unique control from the laparoscopic pool, with surgeon identity required to be identical in every pair to control for operator-related variability. Additional matching variables included ASA class (exact match), BMI within ± 3 kg/m², and age within ± 5 years. Sex was matched when feasible but was not used as an exclusion criterion. This strategy ensured clinically balanced pairs while preserving adequate sample size for comparative analyses.Cases for which no adequate counterpart could be identified were excluded to maintain strict matching fidelity. This approach resulted in two cohorts of 54 well-balanced patients with comparable preoperative risk profiles, allowing an unbiased comparison of operative performance metrics between the Maestro™ and conventional laparoscopic approaches.

Inclusion criteria were adult patients with severe obesity (BMI ≥ 35 kg/m² with comorbidities or ≥ 40 kg/m²), eligible for primary RYGB according to international guidelines.

Statistical Process Control (SPC) charts provide a visual way to track how operative performance evolves over consecutive cases. By separating normal random variation from meaningful process changes, SPC analysis helps determine whether a surgical workflow is stable and consistent, or if special factors are introducing variability.

**Fig. 3:**
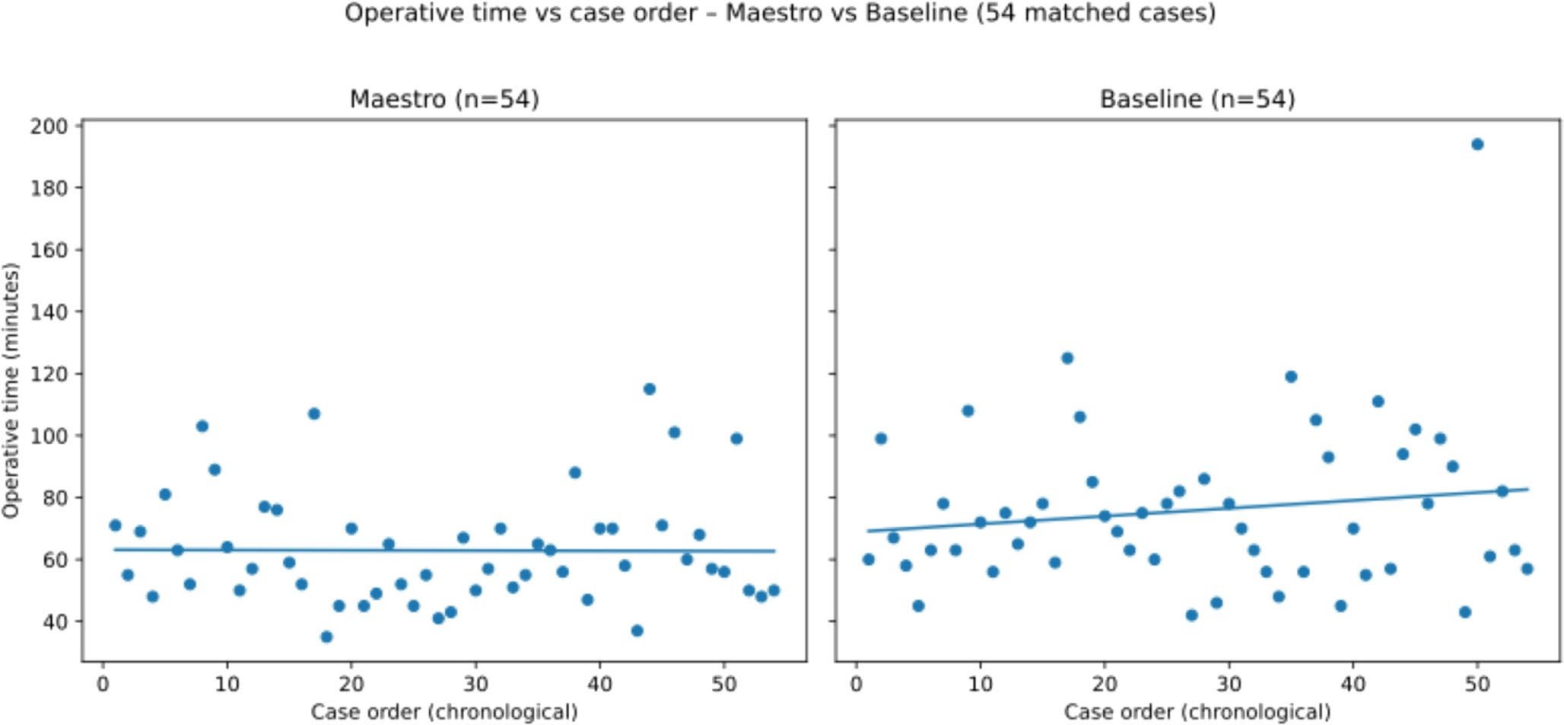
Operative VS case order – Maestro vs Baseline (54 Matched cases)

Operative time (Operative time was defined as skin-to-skin time, measured from initial incision to final skin closure, and did not include anesthesia induction, operating room setup, or postoperative turnover) was plotted sequentially for each cohort to visualize how performance evolved over consecutive cases. Individual control charts were constructed using the group mean and ± 3 standard deviations to identify extreme values and observe overall dispersion across cases.

This analysis was used to describe temporal patterns of operative duration within each group.

For each group, the process mean and the upper and lower control limits (UCL/LCL) were defined as the mean ± 3 standard deviations.

This investigation was conducted within the framework of a post‑market clinical registry evaluating Maestro. The data were derived from a large, multicenter post-market clinical registry including multiple institutions (*N* = 5) and indications. The present analysis was investigator-initiated and conducted independently at a single center, based on a predefined subset of registry cases. The device manufacturer had no role in study design, data analysis, data interpretation, or manuscript preparation.The registry was duly declared to the French Data Protection Authority (CNIL, reference n°223283).

**Fig. 4:**
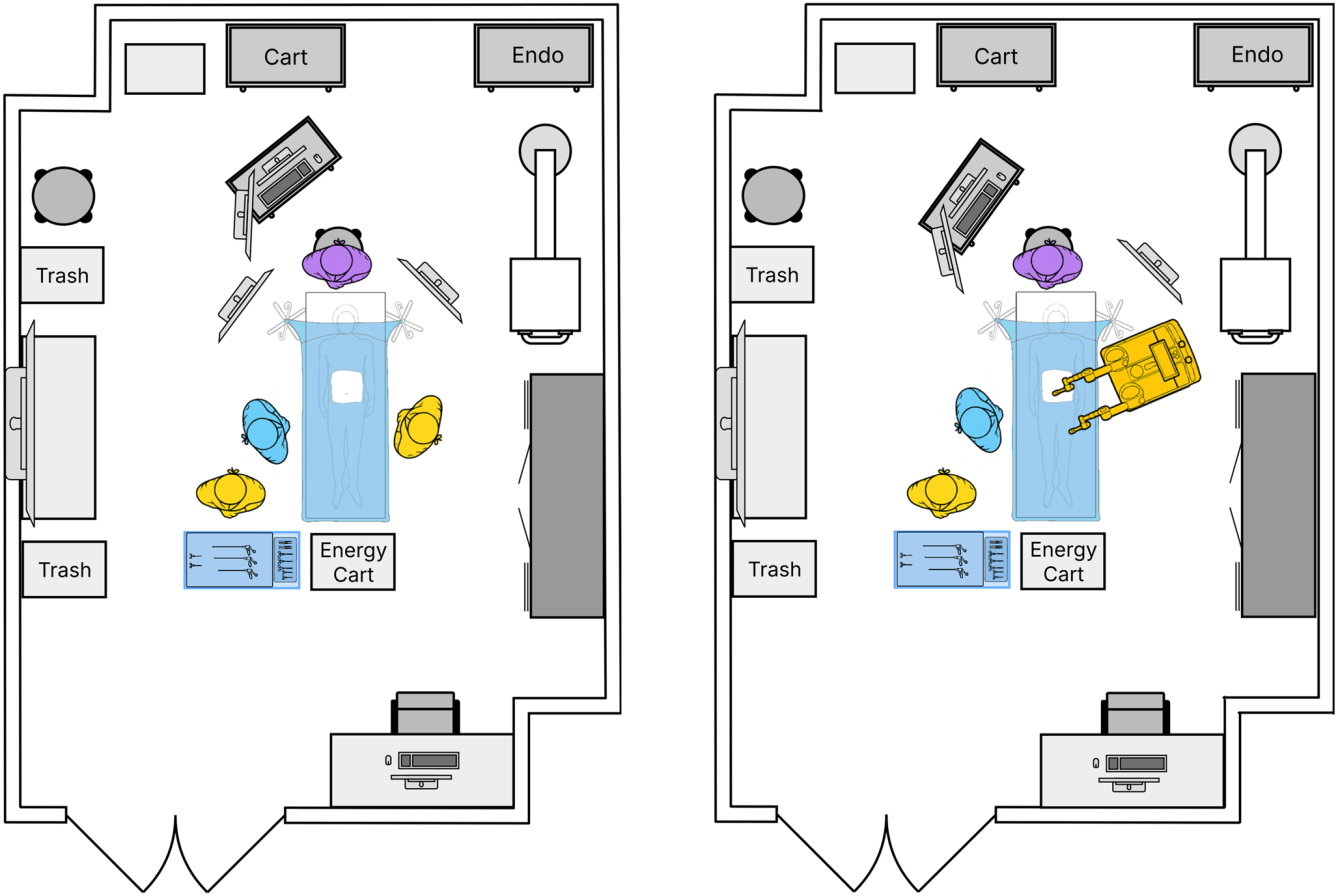
32.5m^2^ (350 Sqft) Operating Room

### The Maestro™

The Maestro™ (Moon Surgical, Paris, France) is a novel, AI-powered surgical platform that gives MIS surgeons enhanced stability, autonomy, and control in minimally invasive surgery (MIS). Unlike master-slave telemanipulated robotic systems, Maestro is fully integrated into the laparoscopic workflow and operated directly by the surgeon at the patient’s bedside. This allows for real-time intraoperative adaptation, preservation of haptic feedback, and full autonomy and control throughout the procedure. Maestro comprises two highly sensitive robotic arms mounted on a compact mobile cart that is positioned near the operating table (Fig. 1). Each arm can support standard 5–10 mm laparoscopic instruments, including the latest generation of laparoscopes and graspers. The arms are powered and offer high-resolution control of positioning and movement with speed and precision. One of the key features of the Maestro system is the ScoPilot™ module—a computer vision-powered algorithm embedded in the system’s edge-computing GPU. This proprietary software uses a computer vision model trained on real surgical videos to recognize laparoscopic instruments in the visual field. The model is trained offline on annotated recordings of real procedures so it can reliably detect the active surgical tool. It enables real-time tracking of the active surgical instruments and automatically adjusts the camera position to maintain optimal framing of the operative field: ScoPilot can follow instruments’ movement and center the image on the target without manual adjustment, significantly reducing the cognitive load for the surgeon and his assistants. This functionality can be activated at the surgeon’s discretion during the procedure. The surgeon remains fully in control at all times, with the ability to adjust or override the camera positioning whenever desired. Maestro was designed for seamless integration into existing OR workflows. It is compatible with laparoscopic towers, cameras, and insufflators, and does not require dedicated robotic instruments or specific trocars. The surgeon remains scrubbed, seated or standing, at the patient’s side, maintaining control of all surgical instruments while freeing up the workspace for their comfort and positioning throughout the surgery. This preserves the familiarity of traditional laparoscopy while eliminating many of the inefficiencies associated with assistant-dependent workflows, and non-ergonomic positioning often encountered in manual laparoscopy.

### Surgical Technique

Both MA-RYGB and L-RYGB were performed using the same five-trocar technique with a standardized antecolic, antegastric Roux-en-Y configuration. The jejunojejunostomy was performed with a 60 mm linear stapler, and the gastrojejunostomy was constructed using a 30 mm linear stapler followed by hand-sewn closure of the enterotomy. In the MA‑RYGB group, Arm 1 of the Maestro system managed visualization provided by the laparoscope, while Arm 2 provided active tissue exposure—such as retracting the stomach, mesocolon or small bowel—serving as a third hand for the surgeon and freeing the surgical assistant to perform other critical intraoperative tasks. When required, retraction of the left hepatic lobe was achieved using a conventional table‑mounted laparoscopic retractor, operated independently of the Maestro™ system. All stapling and suturing were performed manually by the operating surgeon.

## Results

### Patients Characteristics

During the study period, 108 cases were evaluated – 54 MA-RYGB and 54 L-RYGB. As a result of the matching of the two cohorts, both were as follow:

The patients analyzed in the MA-RYGB group had a mean age of 46.4 (SD 9.5) years and a mean BMI of 41.1 (SD 4.6) kg/m^2,^ and a mean ASA of 2.9 (SD 0.2). There were 44 women (81%) and 10 men (19%).

The patients matched in the L-RYGB group had a mean age of 46.4 (SD 10.3) years and a mean BMI of 41.0 (SD 4.7), and a mean ASA of 3.0 (SD 0.1). There were 47 women (87%) and 7 men (13%).

The two principal surgeons each contributed a balanced number of cases to both groups. Full details of the patient demographics are shown in Table 1.

**Table 1:** Patients characteristics

Values	MA-RYGB	L-RYGB	*p*-value
Gender- Male (n, %)	10 (19%)	7 (13%)	> 0.05 (0.60)
Average of Age at Time of Surgery, years (SD)	46.5 (9.5)	46.4 (10.3)	> 0.05 (0.88)
Mean ASA Classification ( Range)	2.94 (0.23)	2.98 (0.14)	> 0.05 (0.32)
Mean Body Mass Index, kg/m2 (SD)	41.07 (4.6)	41.04 (4.7)	> 0.05 (0.84)

### Operative Time

The transition from L-RYGB to MA-RYGB was associated with a 19.3% reduction of operative time. Overall, the mean operative time for MA-RYGB was 62.9 min (SD 18.03), compared to 75.9 (SD 25.97) for L-RYGB (*p* < 0.001).

This difference reflects a reduction in surgical execution time only, as operative time was defined as skin-to-skin and excluded perioperative components.

### Post-operative Outcomes – 30 Day Outcomes

There were 2 (3.7%) post-operative complications in the MA-RYGB and 4 (7.4%) in the L-RYGB group (*p* = 0.68). And no device-related complications or conversions in the MA-RYGB group.

In the MA-RGYB group, one subject experienced a melena episode, likely secondary to eschar sloughing, that resolved after surgical reintervention. Another subject experienced abscess at the gastrojejunal anastomotic site, without fistula formation, managed conservatively with antibiotics and resulting in a favorable outcome. In the L-RYGB group, one patient experienced upper remnant necrosis after conversion of a gastric sleeve to a RYGB for weight regain, requiring laparoscopic partial gastrectomy. Two patients developed gastrojejunal anastomotic leaks, both successfully managed with endoscopic stent placement. Another patient was readmitted for abdominal pain and biological inflammatory syndrome, and recovered favorably under antibiotic treatment.

Full details of intraoperative and postoperative outcomes are shown in Table 2.

**Table 2:** Intraoperative and postoperative outcomes

Values	MA-RYGB	L-RYGB	*p*-value
Mean Duration of Surgical Procedure, min (SD)	62.9 (18)	75.9 (26.0)	< 0.05 (0.00087)
Intraoperative Conversion (n, %)	0 (0%)	0 (0%)	> 0.05
Post-operative Complications (n, %)	2 (3.7%)	4 (7.4%)	> 0.05 (0.68)
Melena Secondary to Eschar Sloughing (n, %)	1 (1.8%)	NA	NA
Abscess at the Gastrojejunal Anastomotic Site (n, %)	1 (1,8%)	NA	NA
Necrosis of the Gastric Remnant (,%)	NA	1 (1.8%)	NA
Gastrojejunal Anastomoic Leak (n,%)	NA	2 (3.7%)	NA
Abdominal Pain (n,%)	NA	1 (1.8%)	NA
Readmissions (n, %)	2 (3.7%)	4 (7.4%)	> 0.05 (0.68)
Unplanned return to OR (n, %)	1 (1.8%)	3 (5.6%)	> 0.05 (0.31)
Device related adverse events	0	NA	NA

### Process Variability and Stability

Statistical process control charts were generated for the 54 matched cases in each cohort, using chronological case order to reflect real-world procedural evolution.

In both groups, operative times were distributed around the mean, with upper and lower control limits (mean ± 3 SD) defining the expected range of variation. Most cases fell within these limits, with only isolated values exceeding them, without evidence of sustained process shifts.

Visual inspection of the SPC charts suggested a more compact distribution and earlier stabilization of operative times in the MA-RYGB cohort compared with conventional laparoscopy (Fig. 2). To formally assess differences in dispersion, an F-test was performed, demonstrating a significantly lower variance in the MA-RYGB group (*p* = 0.009).

Overall, Maestro-assisted RYGB was associated not only with shorter operative time but also with reduced variability, supporting a more consistent operative performance.

To address potential outliers and temporal effects, operative time was plotted against chronological case order for both cohorts (Fig. 3), with cases ordered by operative date. This visualization shows the distribution of individual data points and confirms that operative times were broadly distributed across the study period, with isolated higher values occurring throughout the series rather than clustering at one end. This confirms that the observed reduction in operative time was not driven by a small number of extreme values but reflected a consistent distribution of shorter operative times across cases.

## Conclusions

In this comparative study, the Maestro™ demonstrated a reduction in operative time (*p* < 0.001) for Roux-en-Y gastric bypass (RYGB) by 19.3%.

Postoperative complications occurred in 2 out of 54 (3.7%) Maestro™ cases (3.7%) and 4 out of 54 baseline cases (7.4%) – demonstrating no statistical significance (*p* = 0.68).

The distribution of complication severity (50% Clavien–Dindo II and 50% Clavien–Dindo III in each group) was comparable (*p* = 1.0). No intraoperative complications or device-related errors occurred, and no cases required conversion to open or multiport surgery.

These findings suggest that the use of Maestro™ was associated with a reduction in operative time, while postoperative morbidity appeared comparable between groups.

In addition, the Maestro™ cohort demonstrated reduced variability in operative time (standard deviation 18 vs. 26 min), corresponding to an approximate 30% reduction in dispersion, supported by formal variance testing (*p* = 0.009). Sequential case analysis further suggested a narrower distribution and fewer fluctuations between consecutive cases.

From an operational perspective, although a 13-minute reduction per case may appear modest at the individual level, reduced variability and improved temporal consistency may contribute to more predictable operating room scheduling and workflow efficiency.

MA-RYGB proved safe and non-inferior to standard laparoscopy, achieving a statistically significant reduction in operative time. These findings support the potential of collaborative surgical platforms to standardize laparoscopic workflows while improving surgical efficiency and consistency.

AI-enhanced platforms such as Maestro represent a new frontier in laparoscopic surgery, combining the familiarity of conventional minimally invasive surgery with the precision, stability, and autonomy of robotic systems. This new category of collaborative platforms is designed to elevate rather than replace surgical skills, providing stable, responsive control of the laparoscope and retractors. Compared to large form factor robotic systems that require high capital investment, Maestro’s compact footprint (Fig. 4) and compatibility with existing laparoscopic infrastructure make it highly adaptable for routine use. Its ability to enhance workflow efficiency while maintaining direct bedside control may explain the suggested improved consistency observed in this study.

This study has limitations. It was conducted at a single center, and although cases were matched on key clinical variables, the absence of randomization means that residual confounding factors cannot be excluded. In particular, factors such as prior abdominal surgery or patient sex, although partially addressed through matching when feasible, were not incorporated as strict matching variables and may still represent residual confounders. The procedures were performed by two different surgeons, which may introduce operator-dependent variability; although both had only minimal prior exposure to the Maestro platform before the study (one had performed a single case and the other nine), differences in their individual learning curves may still have influenced the results. Lastly, given the limited number of adverse events, further studies would be necessary to draw conclusions on complications rates.

Despite those limitations, by suggesting that bariatric procedures can be safely and efficiently performed with an advanced minimally invasive surgery (MIS) platform, this study lays the groundwork for re-evaluating current surgical workflows. Maestro™ elevated operative performance during RYGB, yielding shorter operative and in-room times, lower variability, and fewer outliers in overall room duration.

These results are encouraging and dedicated learning-curve analyses (e.g., CUSUM or segmented regression) and further randomized prospective studies are needed to better characterize the impact of Maestro™ on surgical performance and workflow stabilization, and to confirm its role in the evolution of minimally invasive bariatric surgery.

## Data Availability

All data supporting the findings of this study are available within the paper.
